# Detection of differentially expressed genes involved in osteoarthritis pathology

**DOI:** 10.1186/s13018-018-0734-0

**Published:** 2018-03-07

**Authors:** Honglai Tian

**Affiliations:** Department of Orthopaedics, Affiliated Hospital of Shandong University of Traditional Chinese Medicine, No. 16369 Jingshi Road, Lixia District, Jinan City, Shandong 250014 China

**Keywords:** Osteoarthritis, Transcription factor, Meta-analysis, Gene expression profiling datasets

## Abstract

**Background:**

Osteoarthritis (OA) is the most common chronic disorder of joints; however, the key genes and transcription factors (TFs) associated with OA are still unclear. Through bioinformatics tools, the study aimed to understand the mechanism of genes associated with the development of OA.

**Methods:**

Four gene expression profiling datasets were used to identify differentially expressed genes (DEGs) between OA and healthy control samples by a meta-analysis. Gene Ontology and Kyoto Encyclopedia of Genes and Genomes pathway enrichment analyses were performed with Multifaceted Analysis Tool for Human Transcriptome (MATHT). Subsequently, a protein–protein interaction (PPI) network was constructed for these DEGs. Significant network modules were identified using ReactomeFIViz, and the pathway of each module was enriched using MATHT. In addition, TFs in the DEGs were identified.

**Results:**

In total, 690 DEGs were identified between OA and healthy control samples, including 449 upregulated and 241 downregulated DEGs. Additionally, 622 nodes and 2752 interactions constituted the PPI network, including 401 upregulated and 221 downregulated DEGs. Among them, *FOS*, *TWIST1*, *POU2F1*, *SMARCA4*, and *CREBBP* were also identified as TFs. RT-PCR results showed that the expression levels of *Fos*, *Twist1*, *Pou2f1*, *Smarca4*, and *Crebbp* decreased in mice with OA. In addition, *FOS*, *TWIST1*, *SMARCA4*, and *CREBBP* were involved in the positive regulation of transcription from the RNA polymerase II promoter.

**Conclusions:**

*TWIST1*, *POU2F1*, *SMARCA4*, and *CREBBP* may play an important role in OA pathology.

## Background

Osteoarthritis (OA) is the most common chronic disorder of joints, such as knee joints, hip joints, and small finger joints [1]. In China, approximately 10% of the total population experiences OA [2]. The main symptoms of patients with OA are pain, swelling, and joint deformity because of cartilage breakdown [3]. There is no cure due to the long-term nature of the disease and multiple mechanisms associated with it, but physical activity, improving joint mobility and flexibility using assistive devices, and surgery can help improve symptoms [4, 5]. Thus, studies on etiological factors of OA are important.

Many studies have reported on factors that contribute to the development of OA, such as obesity, fracture, surgery or ligament tears, and genes [6, 7]. Various genes make individuals more susceptible to OA. Researchers have found that fatty acid amide hydrolase (*FAAH*) expression is related to increased pain sensitivity and is upregulated in patients with knee OA than in people without OA [8]. Subsequently, *FAAH* inhibitors, such as URB597, PF-04457845, and OL-135, have been focused on for OA treatment [9–11]. In addition, long and short proteins, which are encoded by *DVWA* and related to knee OA susceptibility, are mainly expressed in articular cartilage [12].

Transcription factors (TFs) are proteins that control the rate of transcription in molecular biology, which regulates gene expression [13]. The expression of SAF-1, an inflammation-responsive TF, was found to be overexpressed in moderate-to-severely damaged OA cartilage tissues [14]. Therefore, screening the key genes and TFs associated with OA is important for determining OA pathology.

Through bioinformatics tools, the present study aimed to understand the mechanism of genes associated with the development of OA. Differentially expressed genes (DEGs) were identified using four gene expression profiling datasets, GSE55235, GSE55457, GSE1919, and GSE12021, based on a meta-analysis. Gene Ontology (GO) and Kyoto Encyclopedia of Genes and Genomes (KEGG) pathway enrichment analyses were performed with MATHT (www.biocloudservice.com). Subsequently, a protein–protein interaction (PPI) network was constructed for these DEGs. Significant network modules were identified using ReactomeFIViz, and the pathway of each module was enriched using MATHT. In addition, TFs in the DEGs were identified, and the key DEGs were verified using quantitative real-time polymerase chain reaction (qRT-PCR). These findings may provide a novel understanding of the molecular mechanisms underlying OA.

## Methods

### Data acquisition

Four datasets, GSE55235, GSE55457, GSE1919, and GSE12021, including human OA and healthy control samples were downloaded from the Gene Expression Omnibus (http://www.ncbi.nlm.nih.gov/geo/) database. The characteristics of each dataset are shown in Table 1.

**Table 1:** The characteristic of four datasets

Accession	OA count	Normal count	Platform
GSE55235	10	10	Affymetrix Human Genome U133A Array
GSE55457	10	10	Affymetrix Human Genome U133A Array
GSE1919	5	5	Affymetrix Human Genome U95A Array
GSE12021	20	13	Affymetrix Human Genome U133A Array, Affymetrix Human Genome U133B Array

*OA* osteoarthritis

### Data preprocessing

Raw CEL files were read using the Affy package in R software (version 1.28.0, http://www.bioconductor.org/packages/release/bioc/html/affy.html) [15]. Subsequently, data preprocessing was performed with RMA [16], such as background correction, normalization, and expression calculation. Each probe ID was transformed into a gene symbol. The probes corresponded to gene symbols according to the latest annotation file. If any probe corresponded to multiple genes, its expression value was removed. If more than one probe corresponded to the same gene symbol, the mean of the probe was used as the expression level of the gene.

### Identification of DEGs using a meta-analysis

The MetaDE package in R software was used to integrate the data in the four datasets [17], and the DEGs in OA samples were identified from genes in the control samples. The expression value of each gene on different platforms was evaluated for heterogeneity and unbias, including *τ*^2^ (estimated amount of heterogeneity) and *Q*_*p*val_ (*P* values for the heterogeneity test). If *τ*^2^ = 0 and *Q*_*p*val_ > 0.05, the gene was homogeneous and unbiased. The differential expression of genes was then detected, and only genes with *P* < 0.05 were considered significant. Subsequently, the false discovery rate (FDR) of each gene was calculated using Benjamini–Hochberg correction. Genes with *τ*^2^ = 0, *Q*_*p*val_ > 0.05, *P* < 0.05, and FDR < 0.01 were identified as DEGs. On the basis of these DEGs, the log_2_ fold change (log_2_FC) was calculated for each gene in the four datasets. If log_2_FC > 0, gene expression was upregulated in OA samples; otherwise, it was downregulated in OA samples.

### GO enrichment function and pathway analysis

To determine the DEGs involved in biological processes (BPs), cellular components (CCs), molecular functions (MFs), and pathways, GO and KEGG pathway enrichment analyses were performed with MATHT based on Fisher’s test. A *P* value of < 0.05 was considered significant.

### Construction of the PPI network

The interaction between proteins was analyzed using STRING (version 10.0) for the DEGs using default parameters. The threshold value was required confidence (combined score) > 0.4. Subsequently, the PPI network was visualized using Cytoscape (version 3.2.0, http://www.cytoscape.org/). In the network, the node represents a protein, the line represents the interaction, and the degree represents the number of interactions. Then, CytoNCA in Cytoscape was used to analyze the network topology under the “without weight” condition. The degree centrality, betweenness centrality, and closeness centrality of each node were obtained.

### Module analysis in the PPI network

The ReactomeFIViz app-applied MCL graph clustering algorithm was used to generate a subnetwork for a list of significant network modules [18]. In addition, the average Pearson correlation coefficient among genes involved in the same module was calculated. On the basis of the subnetwork, the pathway in each module was enriched using MATHT.

### Construction of the transcriptional regulatory network

On the basis of the TF–target gene data from the Transcriptional Regulatory Relationships Unraveled by Sentence-based Text Mining website, the TFs in the DEGs were identified. The network was visualized using Cytoscape.

### Animal model of OA

To establish the animal model of OA, male rats were randomly divided into control and model groups (30 rats per group). After acclimatization for 3 days, rats in the control group were given normal food without any other treatment. The left knee joints of rats in the model group were subjected to anterior cruciate ligament transaction, and the right knee joints served as the control [19]. Subsequently, the rats were sacrificed and the joints were harvested at 8 weeks post surgery.

### Verification of qRT-PCR results

To confirm the results, expression levels of *FOS*, *TWIST1*, *POU2F1*, *SMARCA4*, and *CREBBP* were detected using qRT-PCR. Total RNA was extracted from the synovial tissues of rats using TRIzol reagent following the manufacturer’s instructions (TAKARA, Dalian, China) under low temperature. Subsequently, first-strand cDNA was prepared from the RNA obtained from synovial tissues using PrimeScript™RT Master Mix according to the manufacturer’s instructions (RR036A, TAKARA). Rat glyceraldehyde-3-phosphate dehydrogenase (GAPDH) was used as the endogenous control. Primers used for *FOS*, *TWIST1*, *POU2F1*, *SMARCA4*, and *CREBBP* and GAPDH were based on the rat sequences (Table 2). Relative amounts of mRNAs were obtained using the Relative Expression Software Tool.

**Table 2:** The rat sequences of primers used for RT-PCR

Primer	Sequences (5′–3′)
GAPDH-F	AGACAGCCGCATCTTCTTGT
GAPDH-R	CTTGCCGTGGGTAGAGTCAT
FOS-F	GTGACAGCCATCTCCACCAG
FOS-R	TCCTTTCCCTTCGGATTCTC
TWIST1-F	TTTCACAAGAATCAGGGCGTG
TWIST1-R	CCGTTGCCTCTGGGAATCTC
POU2F1-F	GAGCAGCGAGTCAAGATGAGA
POU2F1-R	GGGCTGCTTCTCAAAGTCCA
SMARCA4-F	GGACGCTGTGATCAAGTACA
SMARCA4-R	GGTTTCGGATGCGTTCCTTG
CREBBP-F	CCAGGCAGGTGTTTCACAG
CREBBP-R	ACAGGAGTGGATGGCTGAGT

*F* Forward primer, *R* Reverse primer

### Statistical analysis

The OA and control groups were compared using unpaired Student’s *t* test by SPSS Statistics V22.0 (SPSS Inc., Chicago, IL, USA). *P* < 0.05 was considered significant.

## Results

### Results of function and pathway enrichment

**Fig. 1:**
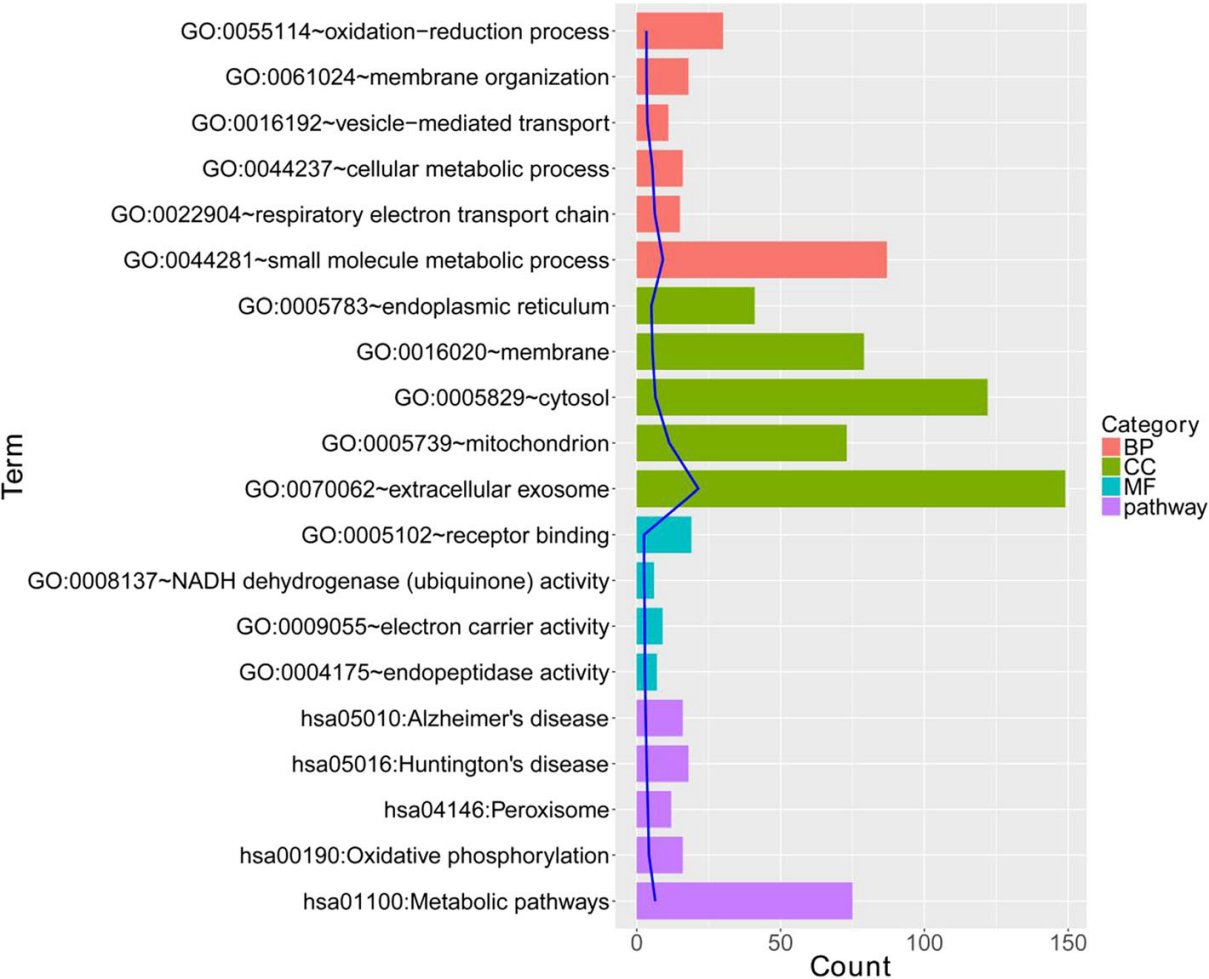
The top five biological process (BP), cellular component (CC), and molecular function (MF) terms and pathways of upregulated differentially expressed genes (DEGs)

**Fig. 2:**
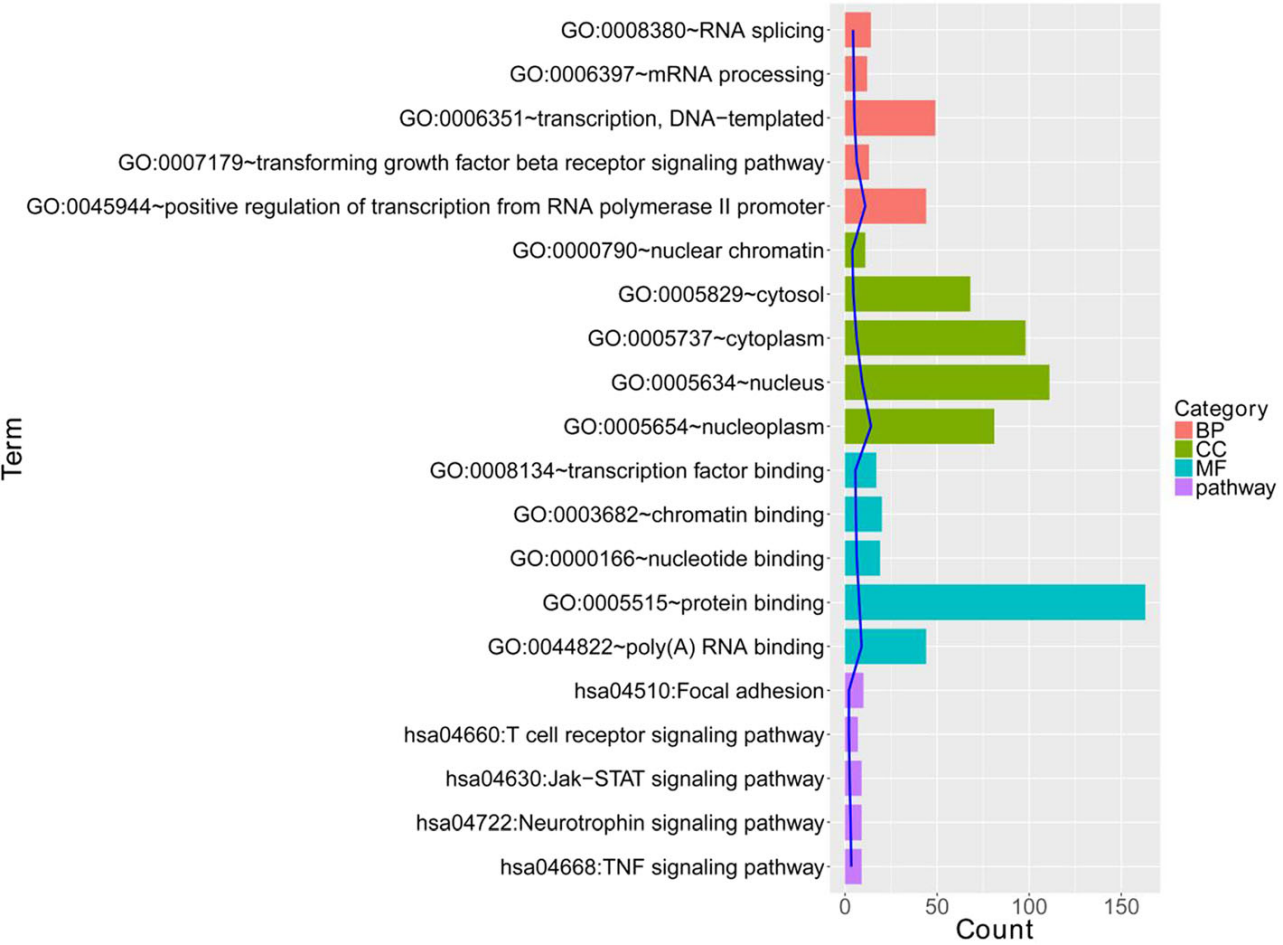
The top five BP, CC, and MF terms and pathways of downregulated DEGs

In total, 690 DEGs were identified between the OA and healthy control samples after the meta-analysis, including 449 upregulated and 241 downregulated DEGs. In the whole upregulated DEGs, 93 BP terms (vesicle-mediated transport, oxidation-reduction process, etc.), 38 CC terms (AP-2 adaptor complex, etc.), 18 MF terms (protein binding, endopeptidase activity, NADH dehydrogenase [ubiquinone] activity, etc.), and 17 pathways (metabolic pathways, oxidative phosphorylation, peroxisome, etc.) were enriched. The top five terms are shown in Fig. 1. In the downregulated DEGs, 85 BP terms (positive regulation of transcription from the RNA polymerase II promoter, transforming growth factor beta receptor signaling pathway, etc.), 14 CC terms (nucleoplasm, nucleus, cytoplasm, etc.), 24 MF terms (poly(A) RNA binding, protein binding, nucleotide binding, etc.), and 20 pathways (TNF signaling pathway, neurotrophin signaling pathway, and Jak-STAT signaling pathway) were obtained. The top five terms are shown in Fig. 2.

### The PPI network

A total of 622 nodes and 2752 interactions constituted the PPI network, including 401 upregulated and 221 downregulated DEGs. The top 10 DEGs with a higher degree are shown in Table 3, such as FOS (degree = 55) and CREB-binding protein (CREBBP) (degree = 48), which are hub proteins. In addition, FOS interacted with CREBBP, TWIST1, and SMARCA4 and CREBBP interacted with SMARCA4 in the PPI network.

**Table 3:** The top 10 of DEGs with higher degree

Node	DC	Node	BC	Node	CC
UBC	420	UBC	298,184.7	UBC	0.736655
JUN	77	JUN	10,611.95	JUN	0.494427
HSP90AA1	75	HSP90AA1	10,195	HSP90AA1	0.491686
MAPK1	60	MAPK1	8585.318	FOS	0.476227
FOS	55	KDM2A	8230.978	MAPK1	0.475498
CDC42	52	IL6	6267.055	CDC42	0.473684
CREBBP	48	CREBBP	5555.968	CREBBP	0.470811
IL6	45	FOS	5334.594	IL6	0.469033
YWHAZ	44	CDC42	5333.35	BCL2L1	0.469033
ACACB	42	CAT	4848.764	YWHAZ	0.466567

*DC* degree centrality, *BC* betweenness centrality, *CC* closeness centrality

### Pathways related to modules

**Fig. 3:**
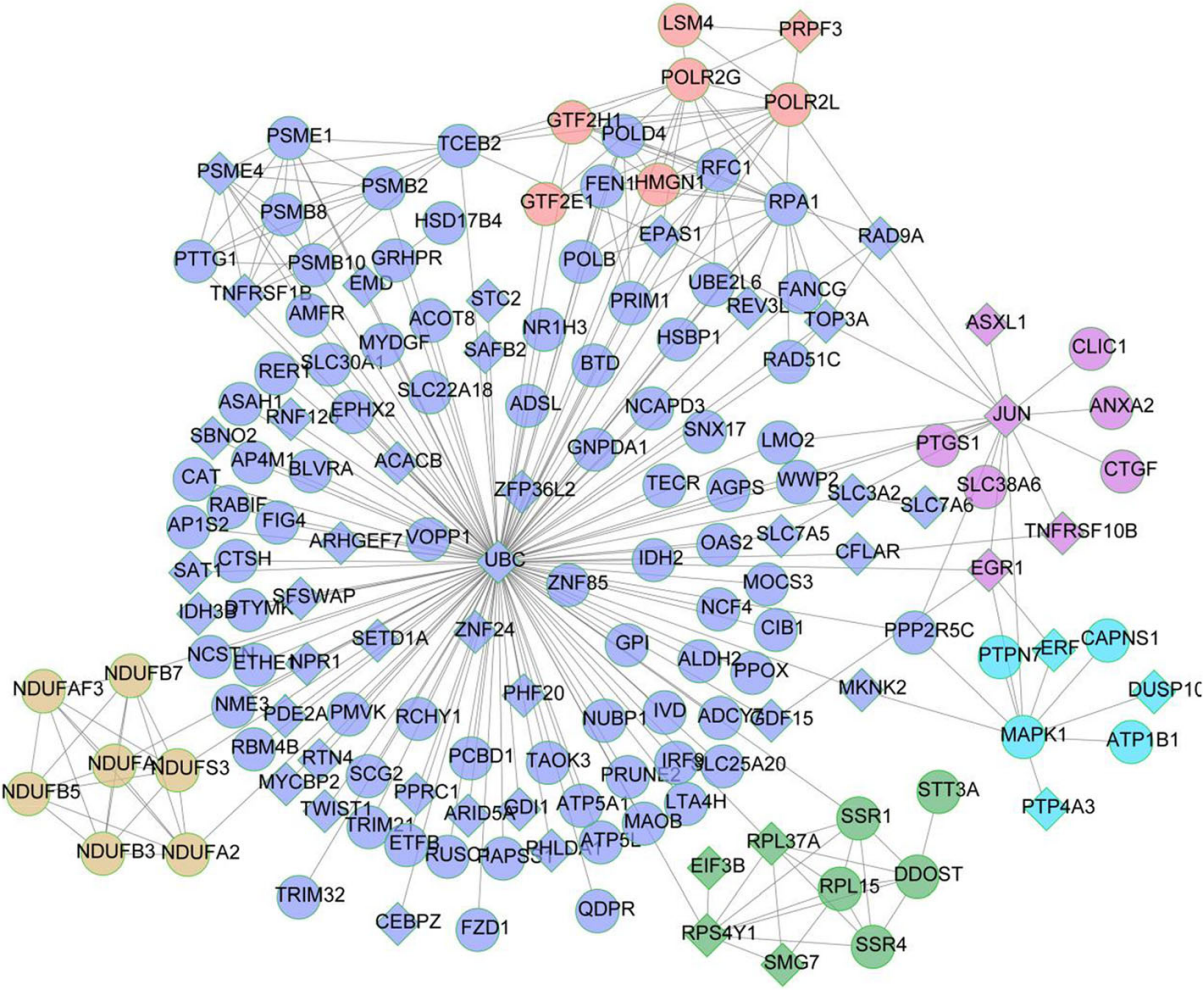
Significant network modules. The circle indicates upregulated DEGs, and the rhombus indicates downregulated DEGs. The different colors indicate different modules

The subnetwork was obtained after ReactomeFI analysis, including 157 nodes (108 upregulated and 49 downregulated DEGs) and 287 interactions, which belong to six different modules (Fig. 3). In addition, five modules were enriched by the following pathways: oxidative phosphorylation, MAPK signaling pathway, and metabolic pathways.

### The transcriptional regulatory network

**Fig. 4:**
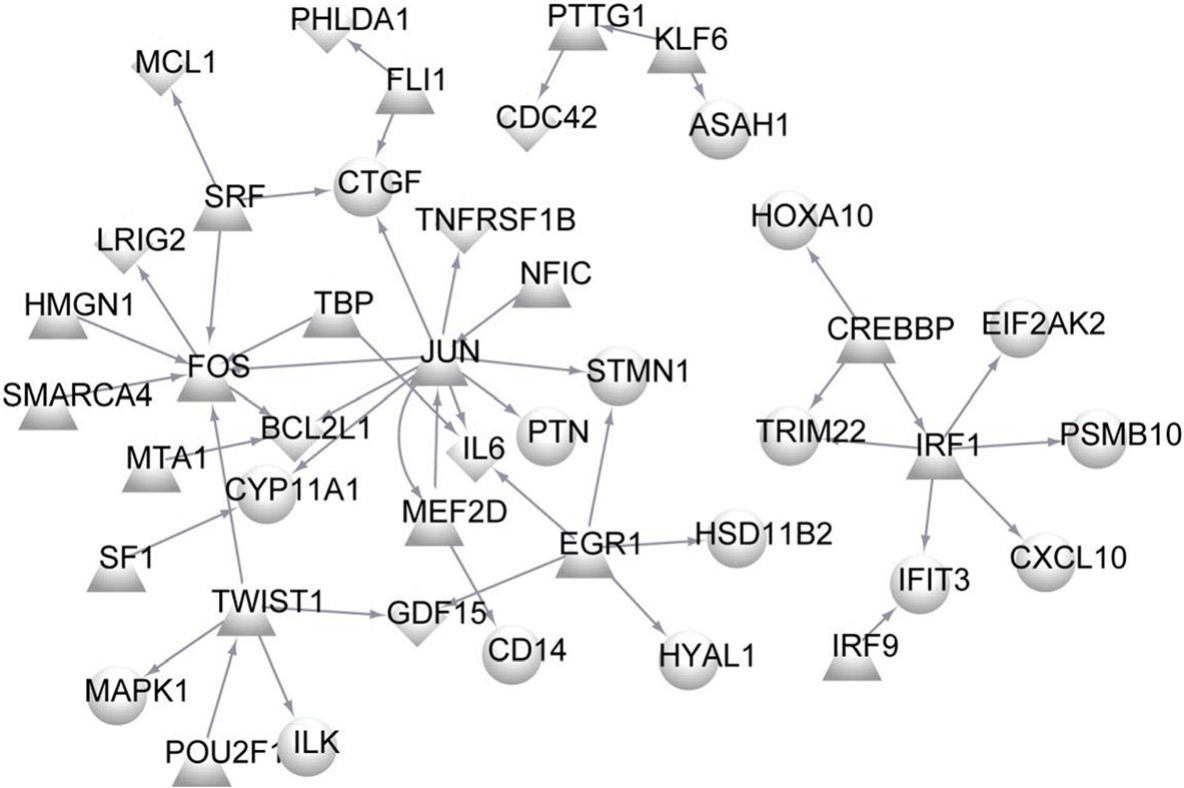
The transcriptional regulatory network. The circle indicates upregulated DEGs, the rhombus indicates downregulated DEGs, and the triangles indicate transcription factors

There were 43 nodes and 47 link pairs in the transcriptional regulatory network, including 19 TFs (such as FOS, TWIST1, POU2F1, SMARCA4, and CREBBP), 16 upregulated DEGs, and 8 downregulated DEGs (Fig. 4).

### Expression levels of candidate genes connected with OA

**Fig. 5:**
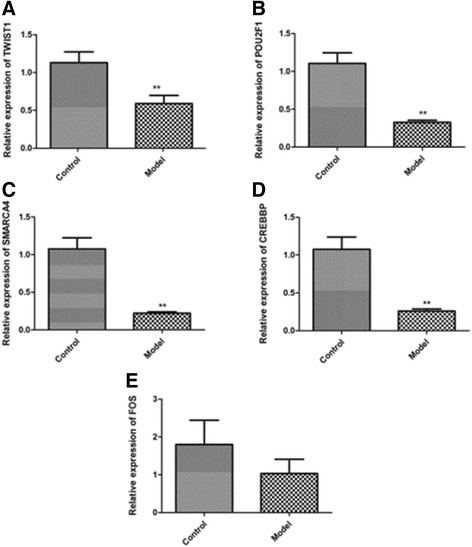
Relative expression levels of *TWIST1* (**a**), *POU2F1* (**b**), *SMARCA4* (**c**), *CREBBP* (**d**), and *FOS* (**e**) in rats with osteoarthritis. *P < 0.05; **P < 0.01

As shown in Fig. 5a, b, the expression levels of *TWIST1* and *POU2F1* significantly decreased in rats with OA (*P* = 0.008), which confirmed the reliability of the bioinformatics method. In addition, the expression levels of *SMARCA4* and *CREBBP* significantly decreased in rats with OA (*P* < 0.001) (Fig. 5c, d). Although *FOS* expression levels decreased in rats with OA, they were not significantly different (*P* = 0.307) (Fig. 5e).

## Discussion

In this study, downregulated DEGs, such as *FOS*, *TWIST1*, *POU2F1*, *SMARCA4*, and *CREBBP*, were identified as TFs. RT-PCR showed that the expression levels of *Fos*, *Twist1*, *Pou2f1*, *Smarca4*, and *Crebbp* decreased in mice with OA. In addition, *FOS*, *TWIST1*, *SMARCA4*, and *CREBBP* were involved in the positive regulation of transcription from the RNA polymerase II promoter. In the PPI network, FOS interacted with CREBBP, TWIST1, and SMARCA4 and CREBBP interacted with SMARCA4.

*TWIST1* encodes a basic helix–loop–helix TF that plays an important role in osteoblast metabolism and differentiation [20]. *TWIST1*, as a critical regulator of osteoblast differentiation in OA pathology, was also identified from the trabecular bone of patients with end-stage OA [21]. In addition, *TWIST1* expression decreased in OA patients and was correlated with the inhibition of normal mineralization in OA patients [22]. Similarly, *TWIST1* expression was downregulated in synovial tissues in OA. In a previous study, the downregulated gene *TWIST1* was a target of the WNT signaling pathway [23], which is related to bone remodeling and pathologies such as OA [24]. Therefore, the downregulated gene *TWIST1* is a key regulator in OA development. *POU2F1* (also known as OCT1) downregulation facilitates osteosarcoma tumorigenesis [25]. Although *POU2F1* has not been reported in OA pathology, it interacts with adenomatous polyposis coli, which negatively regulates the WNT pathway [26, 27]. In addition, *POU2F1* can regulate *TWIST1* expression in the transcriptional regulatory network. Therefore, *POU2F1* might also be a candidate gene connected with OA pathology.

*TWIST1* can regulate *FOS* expression in the transcriptional regulatory network, and *TWIST1* and *FOS* interact with each other in the PPI network. Kinne et al. found that compared to patients with OA or normal joints, c-fos was highly expressed in the synovial membrane of patients with rheumatoid arthritis [28]. In addition, c-fos expression was detected in the superficial layer of cartilage only in 20% of OA patients [29]. A small molecule, harpagoside, as a therapeutic for preventing OA development, can inhibit IL-6 expression by blocking the expression of *c-FOS* in primary human OA chondrocytes [30]. However, FOS expression decreased in our study, probably because the sample sizes and patients from different countries in the present study were not the same as in previous studies. In the transcriptional regulatory network, *SMARCA4* as a TF that modifies *FOS* was also downregulated in OA patients. Besides, *FOS* interacted with *CREBBP* (a hub gene) and *SMARCA4* and *SMARCA4* interacted with *CREBBP* in the PPI network. As reported, the upstream regulator *SMARCA4* interacted with Nur77, which modulates inflammatory gene expression in the transcriptome of bone marrow-derived macrophages [31]. In samples from mice with OA, *Smarca4* and *Crebbp* expression decreased compared to that in control samples. Therefore, *SMARCA4* and *CREBBP* are candidate genes involved in OA pathology.

## Conclusions

*TWIST1*, *POU2F1*, *SMARCA4*, and *CREBBP* may play an important role in OA pathology. Although the regulating interactions among them were obtained from bioinformatics analysis and require further validation, the results provided a guideline underlying the molecular mechanisms of OA and found a novel therapeutic target.
